# A Mediation Model of Food Literacy: A Potential Relationship between Body Image Dissatisfaction and Body Mass Index

**DOI:** 10.3390/medicina60081196

**Published:** 2024-07-24

**Authors:** Neslihan Arslan, Feride Ayyıldız, Kübra Esin

**Affiliations:** 1 Department of Nutrition and Dietetics, Faculty of Health Sciences, Erzurum Technical University, 25050 Erzurum, Türkiye; akdeniz.neslihan91@gmail.com; 2 Department of Nutrition and Dietetics, Faculty of Health Sciences, Gazi University, 06490 Ankara, Türkiye; 3 Department of Nutrition and Dietetics, Faculty of Health Sciences, Tokat Gaziosmanpaşa University, 60250 Tokat, Türkiye; kubra.esin@gop.edu.tr

**Keywords:** obesity, body image dissatisfaction, food literacy

## Abstract

*Background and Objectives*: The global rise in obesity presents a significant public health challenge, with Turkey exhibiting one of the highest obesity rates in Europe. Body image dissatisfaction (BID) and lower food literacy (FL) have been associated with obesity, yet their interplay remains underexplored. This study aimed to investigate the relationship between body mass index (BMI), body image dissatisfaction, and FL in adults in Turkey. *Materials and Methods*: In total, 759 women and 419 men aged 18–64 years old were included in this study. The mean age was 31.34 ± 11.92. A total of 1178 participants completed an online questionnaire assessing anthropometric measurements, BID, using the Stunkard Figure Rating Scale, and FL, using a validated questionnaire. The data were analyzed using descriptive statistics, correlation analysis, and mediation analysis to explore the relationships between variables in SPSS 24.0. *Results*: Women desired to be thinner more frequently than men, and those with negative BID were predominantly individuals with overweight or obesity. Moreover, those with higher FL scores were more likely to be satisfied with their bodies. Correlation analysis demonstrated a negative relationship between BMI and FL (r = −0.94; *p* = 0.001) and a positive relationship between BMI and BID (r = 0.628; *p* < 0.001). Mediation analysis revealed that FL mediated the relationship between BID and BMI (β = −2.281; lower limit = −3.334, upper limit = −1.228). *Conclusions*: The findings highlight the importance of addressing BID and enhancing FL to mitigate obesity risk factors. This study contributes to understanding the complex interplay between BID, FL, and obesity, providing insights for public health interventions aimed at obesity prevention and management.

## 1. Introduction

The World Health Organization (WHO) has declared obesity, which is increasingly becoming a more serious problem than malnutrition, as the most significant global chronic health problem in adults [1]. According to the predictions of the World Health Organization, 167 million adults and children will reach a less controlled level in 2025 due to overweight or obesity [2]. According to the 2017 data of the Turkey Nutrition and Health Survey (TBSA), 34.0% of individuals over the age of 15 are overweight and 31.5% are obese [3]. The increasing prevalence of obesity worldwide increases the burden of mortality and morbidity. According to World Health Organization (WHO) 2022 data, while the prevalence of obesity in Europe is 23.3%, this rate is 32.1% in Turkey [4]. Obesity is caused by a combination of various factors, such as eating disorders, body image dissatisfaction [5], and lower food literacy (FL) [6].

Grogan defines body image as “a person’s perceptions, thoughts and feelings about his or her body” [7]. The concept of body image is intricate, multidimensional, and dynamic, encompassing an individual’s understanding, emotions, and ideas around their physical appearance. It has been commonly established that body image disturbances, including body image dissatisfaction and body image mis-perception, are associated with eating disorders, disordered eating patterns, and inappropriate weight control practices [8]. Body image dissatisfaction (BID) is described by people’s unfavorable attitudes and opinions about their physical appearance. It arises from the difference between the self-perception of one’s body and their ideal body shape [7,9]. Influenced by cultural, social, neurological, and psychological factors, as well as cognitive beliefs, BID poses a significant risk factor for obesity, eating disorders, and psychological issues [9,10,11]. Notably, the DSM-5 lists body image disturbance as a primary psychopathological symptom for the diagnosis of anorexia nervosa, although it can occur in any eating disorder [12]. Body image dissatisfaction is a significant contributing factor to the development of eating disorders and obesity [13,14,15].

The relationship between weight status and BID is complicated [16]. Body image encompasses not only one’s satisfaction or dissatisfaction with their physical appearance, but also a cognitive component that reflects the relationship between the evaluation of body shapes and the positive or negative attitude [17]. People who are obese or overweight are more likely to be subjected to negative evaluations of their bodies [18]. BID may be affected by obesity via psychological distress, such as low self esteem and depression [5]. In a study, it was shown that there was a mediating effect of BID in the relationship between depression/self esteem and body weight [19].

The impact of social interactions on body image is substantial, even though it is a subjective psychological phenomenon. Western societies have recently been promoting a slender appearance, and the media’s portrayal of the ideal female or male body is widely believed to have a significant impact on how individuals assess and perceive their own physical appearance [16]. The majority of women have expressed a desire to lose weight in recent studies [20,21], which is mostly influenced by the portrayal of a slender ideal in the media. On the contrary, men are often more willing to gain weight due to the thought of gaining muscle [22,23]. Women have more tendency to perceive themselves as being more overweight/obese than men [24,25]. There is a possibility that this is because females are more likely to be interested in investigating information regarding nutrition facts like FL [26].

Over the last 25 years, FL has grown in importance in food and nutrition research [27]. Food literacy has arisen to define the day-to-day practicality of healthy eating [28]. While FL lacks a precise definition, it broadly refers to proficiency in food-related skills and knowledge [29]. Food literacy is the set of inter-related information, skills, and behaviors (components) necessary to achieve a healthy diet, defined under the four domains of plan and manage, select, prepare, and eat [28]. It has been shown that individuals with high food literacy tend to consume less salt [30]. Compared with participants with lower levels of food literacy, those with greater levels of food literacy reported a significantly higher frequency of fruit, vegetable, and fish consumption [31]. Furthermore, FL can influence people’s behavior and consumer choices [6]. It has been found in Korea that people with obesity have lower FL [32]. Although research on the relationship between BID and FL is limited, studies have explored connections between BID and healthy eating behaviors [33,34]. A systematic review concluded that adolescents who desire to gain weight have unhealthier dietary habits, and those who want to lose weight have healthier dietary habits [35]. It was determined that female individuals had higher FL than male individuals [27].

Therefore, in this study, we aimed to investigate the relationship between body image dissatisfaction, food literacy, and body mass index, according to gender.

Based on the studies given above, the hypotheses of this study are as follows:

**H1.**  *Body image dissatisfaction is associated with body mass index*.

**H2.**  *Food literacy is associated with body mass index*.

**H3.**  *There is a difference between genders in terms of body image dissatisfaction and food literacy*.

**H4.**  *Food literacy has a mediator effect on body image dissatisfaction and body mass index*.

## 2. Materials and Methods

### 2.1. Research Methodology

**Figure 1:**
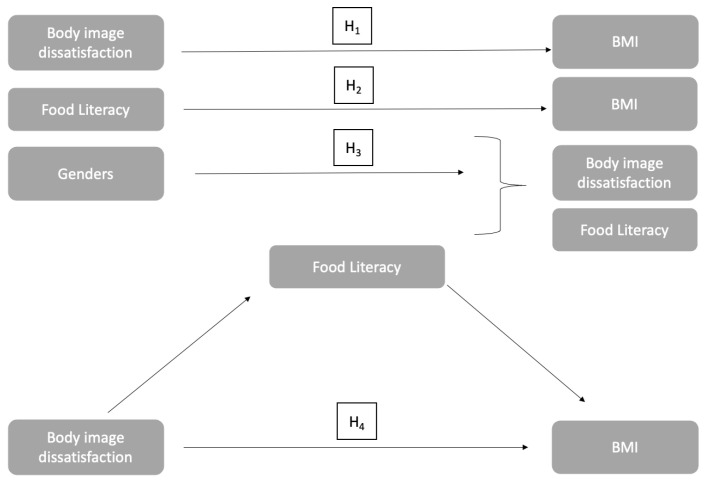
Research model.

The research model given in Figure 1 was developed with the hypotheses established by examining the information in the literature. In this study, we first examined whether there was a difference between the variables in terms of gender and body image dissatisfaction. Then, the relationships between BID, FL, and BMI were analyzed. Finally, FL was used as the mediator, BID as the independent variable, and BMI as the dependent variable.

### 2.2. Study Design

This study was planned and conducted to examine the relationship between FL, body image dissatisfaction, and obesity in women and men aged 18 and over. Researchers made advertisements using social media tools such as Instagram and WhatsApp. The sample number of this study was determined by the snowball sampling method. This study was conducted on 1178 people aged 18–64 who agreed to participate. Healthy and volunteer individuals were included in this study; individuals diagnosed with eating disorders were excluded. Despite being volunteers, individuals who were under the age of 18 and individuals who still needed to complete the survey were excluded from the study. This questionnaire was administered online and consisted of general information (age, education level, working status, economical level, marital status, tobacco use, and alcohol use), anthropometric measurements, the Stunkard Figure Rating Scale, and FL questionnaires.

### 2.3. Anthropometric Measurements

The self-reported body weights and heights were obtained from the participants. Body mass index (BMI) was calculated as weight (kg)/height (m^2^), and subjects were classified according to WHO classification [36]. The obtained BMI values were classified as underweight (≤18.5 kg/m^2^), normal (18.5–24.99 kg/m^2^), overweight (25–29.99 kg/m^2^), and obesity (≥30 kg/m^2^).

### 2.4. Stunkard Figure Rating Scale

The Stunkard Figure Rating Scale (FRS), developed by Stunkard et al., was used to calculate body image dissatisfaction [37]. The participants were asked to choose a male or female silhouette that matched their body. They had to choose their perceived body image (how they saw themselves) and ideal body image (how they desired to look). It was used to calculate a discrepancy score as a measure of BID by subtracting the desired body size score from the existing body size score. Thus, negative or positive scores represent people’s impression of themselves as being more overweight or thinner than their body size. In contrast, a zero score shows body size satisfaction. This approach of determining the degree of body dissatisfaction is a tool for determining how people see their bodies. The positive BID scores were categorized as “desire to be heavier”, negative BID scores were categorized as “desire to be thinner”, and zero BID scores were categorized as “satisfied” [13].

### 2.5. Food Literacy Questionnaire

A short FL questionnaire for adults (SFLQ) created by Krause et al. is a 12-question survey with four- and five-point Likert types. Likert scales were used that offered the following choices: very bad to very good, disagree strongly to agree strongly, very difficult to very easy, very hard to very easy, and never to always [27]. A higher score indicates better FL. Points equal to or higher than 31 indicates excellent; those lower than 31 indicate limited FL. The reliability and validity of the SFLQ scale in Turkish were studied by Durmuş, Gökler, and Havliolu [38]. Cronbach’s alpha score on the FL questionnaire was 0.803.

### 2.6. Statistical Analysis

All analyses were performed using IBM SPSS Statistics version 24.0 for Windows (Statistical Package for the Social Sciences, New York, NY, USA). Descriptive characteristics were presented for all participants of both sexes. After verifying that the continuous variables were normally distributed with the Shapiro–Wilk test and a probability–probability (PP) plot, descriptive characteristics were presented as the mean and the standard deviation. In contrast, qualitative variables were presented as the frequency or median. The chi-squared test, *t*-test for independent variables (means), and Mann–Whitney U test (medians) were used to examine differences between the sexes, and the *p*-values of all of the tests were calculated. “ANOVA” test method was used to compare three or more independent groups. Correlation analysis was conducted using the Pearson (parametric data) or Spearman (nonparametric data) correlation tests, depending on the characteristics of the associated variables. The absolute values of the Pearson and Spearman correlation results were interpreted as very strong for values between 0.9 and 1, strong between 0.7 and 0.89, moderate between 0.40 and 0.69, weak between 0.10 and 0.39, and insignificant between 0 and 0.10 [39].

The mediated structural model analysis was performed to evaluate the effect of BID on BMI by FL. PROCESS SPSS macro v4.0 by Andrew Hayes was used in the mediated structural analysis [40]. When the upper and lower bounds of the bias-corrected 95% confidence intervals (CIs) did not include zero, the effects were evaluated using bootstrap confidence intervals that were corrected for bias and found to be significant. Mediation was assessed by the indirect effect of X (independent variable) on Y (dependent variable) through M (the mediator), which can be significant regardless of the significance of the total effect (the effect of X on Y) and the direct effect (the effect on Y when both X and M are included as predictors). The Hayes index of moderated mediation was used to test moderated mediation.

## 3. Results

The general characteristics of the population are given in Table 1. The mean age of the individuals was 31.34 ± 11.92. It has been observed that there are statistical differences between the education level, employment status, economic status, marital status, and smoking status of male and female individuals (*p* < 0.05). It has been found that female individuals have lower body weights than male individuals, and there is a statistical difference between their BMI classifications (*p* < 0.05). When the food literacy scores of male and female individuals were examined, it was determined that female individuals had higher scores (*p* < 0.05).

The evaluation, FL, and anthropometric measurements of individuals according to their BID classification are given in Table 2. According to BID classification, it was seen that 66.2% of the individuals who wanted to be thinner were women, and 33.8% were men. In the analysis of the whole sample, it was seen that women wanted to be thinner more than men. When the average body weight of the individual was examined, it was determined that those who desired to be heavier were 74.97 ± 15.48 kg, those who were satisfied were 65.07 ± 45.58 kg, and those who desired to be heavier were 62.23 ± 18.99 kg (*p* < 0.001).

When the body mass indexes of the individuals were examined, it was seen that the mean BMI of individuals who wanted to be heavier was 20.70 ± 3.34 kg/m^2^, the average of those who were satisfied was 23.04 ± 11.65 kg/m^2^, and the average of those who wanted to be thinner was 26.51 ± 6.14 kg/m^2^ (*p* < 0.001). It was also determined that those who desired to be thinner consisted mainly of individuals with overweight and individuals with obesity, while those who desired to be heavier consisted mainly of underweight individuals and individuals with a BMI in healthy ranges. Those who were satisfied had a more normal BMI. When the FL of individuals was examined, it was seen that those who were satisfied with their bodies had a higher FL score than the other two groups (*p* < 0.001).

The correlation between BMI, BID, and FL was assessed. Body mass index showed a negative relationship with FL (r = −0.94 *p* = 0.001) and a positive relationship with BID (r = 0.628 *p* < 0.001).

**Figure 2:**
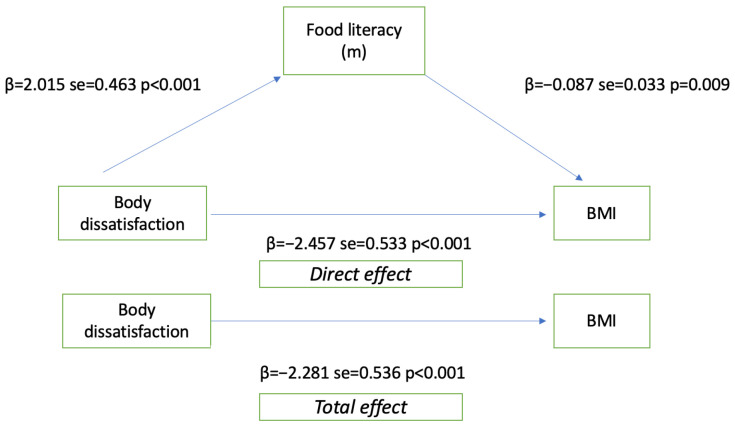
Graphical illustration of mediation analysis.

The effect of FL on body dissatisfaction and on body mass index is given in Table 3 and Figure 2. The effect is significant when the bootstrap indirect effect confidence interval does not contain the upper and lower values 0. The effect of BID on FL is significant (β = 2.01; lower limit = 1.1064, upper limit = 2.9235). BID also significantly affects BMI (β = −2.45; lower limit = −3.504, upper limit = −1.410). In this case, it was concluded that FL is a mediator variable. When FL is added to the model as a mediator, the model becomes significant. When the indirect effect results regarding the mediating effect of FL were examined, it was seen that the lower and upper limit values in the 95% resentment range did not contain 0 values. The mediating role of FL on body image dissatisfaction and body mass index is significant (β = −2.281; lower limit = −3.334, upper limit = −1.228).

## 4. Discussion

In the present study, we aimed to investigate the relationship between body image dissatisfaction, food literacy, and body mass index according to gender. This study’s major findings can be summarized as follows: Female individuals have a lower body weight than male individuals, and there is a statistical difference between their BMI classifications (*p* < 0.05). Female individuals showed higher levels of FL (*p* < 0.05). Gender disparities are evident in body image dissatisfaction, with women exhibiting a notably higher desire to lose weight than men (*p* < 0.05). Individuals experiencing body dissatisfaction, both in desiring weight loss and weight gain, tend to exhibit lower levels of FL (*p* < 0.05). Correlation analysis revealed that BMI is negatively associated with FL and positively associated with BID (*p* < 0.05). Moreover, we have found that FL is a significant mediator between BID and BMI (*p* < 0.001).

Obesity is a significant public health problem [41] and is associated with a number of variables, such as eating disorders, body image [5], and FL [32]. The relationship between body weight and BID is complex. In the present study, the majority of people who desired to be thinner had overweight or obesity, whereas the majority of those who desired to be heavier were underweight or normal. Also, it was shown that the BMIs of individuals who were satisfied with their bodies were more normal. A growing number of research findings indicate that obesity is linked to poor body image, especially in women with obesity and overweight who display higher levels of body image dissatisfaction (BID) than women who have normal weight ranges [5]. Females have lower body weights than males, and there is a statistical difference between the BMI classifications in the present study (*p* < 0.05). Considering that greater body weight and shape are perceived as socially unfavorable, particularly for females, BMI could potentially serve as an indirect biological factor in the development of negative body image [42]. Improving psychological and environmental mediators, which are determinants of obesity, is very important in terms of preventing obesity and thus improving health [43]. Consistent with the existing literature [5,8,44,45], women tended to report higher rates of desire to be thinner compared to men in this study (*p* < 0.05). Women are generally subjected to more sociocultural norms in terms of body image [46]. Self-esteem, personality qualities, and mental health are all important psychological aspects in determining body image beliefs [47]. Women who are suffering higher levels of psychological distress may be more susceptible to negative body image impressions, adding to the documented sex differences in BID [48]. Men tend to be more content with their bodies, perceive themselves as more attractive, and feel they are less overweight compared to women [49]. In a study where the majority of men were overweight, and most women were of average or low weight, the men tended to perceive themselves as lighter than they actually were, while the women perceived themselves as heavier than their actual weight [50]. Recently, Western societies have tended to idealize thinness, and social media has a big impact on ideals for women’s and men’s bodies. While the main focus in women is related to being thin, men usually want to be muscular rather than thin [51].

Despite limited evidence, healthy eating behaviors have been associated with improved body image [33,34], and the potential influence of health literacy on BID has been shown in a study [52]. In the present study, females showed higher levels of FL (*p* < 0.05). There are also some outcomes in terms of gender and FL. A study found that women are more likely to read the nutrition facts on food labels [53]. In another study conducted in Japan, researchers found that females significantly have higher nutrition knowledge than males [54]. In a study, similar to our study, nutritional literacy was found to be higher in women [55]. Improving food literacy rates for male individuals is very important in terms of improving health outcomes [56]. Therefore, it is necessary to develop programs that will contribute to increasing the FL levels of male individuals. Intervention studies with such programs have been found to increase individuals’ confidence in healthy eating and healthy cooking.

In accordance with research, there is a positive relationship between FL, health, and well-being [27,57], and those with higher levels of FL are more likely to eat a balanced and healthy diet [58], which may improve their BMI. Having a higher level of food and nutrition literacy provides individuals with the essential knowledge and skills to effectively navigate the complex food environment. Food literacy and nutrition literacy have been recognized as crucial elements in promoting and sustaining good eating habits [59]. Food literacy may help us to understand the nutritional composition of foods, portion control, and making smart food choices, which can all help with weight maintenance or loss, depending on personal health objectives [60]. Inadequate FL may prevent people from understanding how dietary factors influence disease risk [61]; hence, increasing FL could potentially decrease obesity risk within the general public. The association between FL and BMI has been shown in various studies [6,32,62]. However, there are also studies that have failed to find the relationship between nutrition knowledge and BMI [63,64,65]. Interestingly, it has been found that nutrition literacy is positively associated with BMI [54]. When we evaluate the relationship between FL and body mass index, there is a negative relationship between them. In the mediation analysis, FL was found to be a mediator for BID and BMI. In our study, individuals experiencing body dissatisfaction, both in desiring weight loss and weight gain, tend to exhibit lower levels of FL (*p* < 0.05). These results in our study can be attributed to the fact that it is known that as FL increases, healthy nutrition will increase [58]. As healthy nutrition increases, having a healthy body weight will increase, and obesity will decrease.

In a conducted study, better nutritional literacy with intervention is associated with weight loss in adults [66]. In the previous nutrition intervention study, the authors aimed to increase food literacy and improve dietary behaviors. It was concluded that developing and sustaining food literacy knowledge and behaviors that are essential for healthy food selection and preparation should result in improved diet quality and, ultimately, health outcomes [67]. In future intervention studies on FL, it will be useful to develop programs considering the type of intervention and target age.

Our limitations include the self-reported recording of body weight and height, which may be biased. However, in studies comparing BMI calculated from self-reported body weight and height with measured BMIs, high correlations were observed [68,69].

Another limitation is using body mass index as the only indicator of obesity. The fact that our study is a cross-sectional study can be considered a limitation in terms of not being able to see the cause-and-effect relationship. The fact that this study was conducted on 1178 individuals is thought to reflect the Turkish sample. However, this study population consisted of Turkish citizens. Therefore, the findings cannot be generalized to broader populations.

Our other strengths include the inclusion of both women and men in the study, the similarity of age groups between women and men, and the inclusion of individuals with various distributions of body mass index. In addition, this study is the first to evaluate BID and FL, a factor associated with obesity, together.

## 5. Conclusions

This study conducted in adults reaffirms that women have more negative body image and highlights the significant association between BMI, BID, and FL. Moreover, FL emerges as a critical mediator between BID and BMI. The importance of body image and FL in preventing obesity is emphasized in this study. Improving body image and increasing FL can be added to strategies for preventing obesity in society. Intervention studies on BID and FL should be planned for future studies, and the cause-and-effect relationship should be revealed. Raising food literacy in individuals who are obese or overweight might contribute to improvements in their food skills, healthier dietary behaviors, and body weight. The findings suggest that public health promotion practitioners and policy makers prioritize the implementation of new public health policies that specifically aim to enhance food literacy among the general population.

## Figures and Tables

**Table 1:** The evaluation of general characteristics, food literacy, anthropometric measurements, and body image dissatisfaction, according to gender.

	Male (n = 419)	Female (n = 759)	Statistical Analysis
	n (%)	n (%)	
Age (years)	35.73 ± 12.77	28.92 ± 10.73	*p* < 0.001
Education Level			*p* < 0.001
Literate	1 (0.2)	9 (1.2)	
Primary School	12 (2.9)	24 (3.2)	
Middle School	33 (7.9)	17 (2.2)	
High School	54 (12.9)	108 (14.2)	
Bachelor	233 (55.6)	541 (71.3)	
Post graduate	86 (20.5)	60 (7.9)	
Working Status			*p* < 0.001
Yes	275 (65.94)	250 (32.9)	
No	144 (34.06)	509 (67.1)	
Economical Level			*p* < 0.001
Income more than expenditure	123 (29.4)	134 (17.7)	
Income equal to expenditure	203 (48.4)	361 (47.6)	
Income lower then expenditure	93 (22.2)	264 (34.8)	
Marital Status			*p* < 0.001
Single	196 (47)	500 (65.9)	
Married	223 (53)	259 (34.1)	
Tobacco Use			*p* < 0.001
Yes	158 (37.7)	151 (19.9)	
No	213 (50.8)	577 (76.0)	
Old user	48 (11.5)	31 (4.1)	
Alcohol Use			*p* = 0.717
Yes	64 (15.3)	110 (14.5)	
No	355 (84.7)	649 (85.5)	
Body weight (kg)	82.82 ± 13.57	64.29 ± 30.13	*p* < 0.001
BMI (kg/m^2^)	27.02 ± 9.07	23.73 ± 6.93	*p* < 0.001
BMI classification	6 (1.43)	77 (10.1)	*p* < 0.001
Underweight	137 (32.85)	426 (56.1)	
Normal	188 (45.08)	183 (24.11)	
Overweight	86 (20.62)	73 (9.61)	
Obese	6 (1.43)	77 (10.1)	
FL total score	31.94 ± 6.37	34.65 ± 6.94	*p* < 0.001

BID: Body image dissatisfaction, BMI: body mass index, FL: food literacy.

**Table 2:** The evaluation of gender, anthropometric measurements, body mass index, and food literacy, according to body image dissatisfaction.

	Desire to Be Heavier (BID ≥ 1) (n = 156)	Satisfied (BID = 0) (n = 286)	Desire to Be Thinner (BID ≤ 1) (n = 736)	*p*
	n (%)	n (%)	n (%)	
Gender				*p* < 0.001
Women	78 (50)	194 (67.8)	487 (66.2)	
Men	78 (50)	92 (32.2%)	249 (33.8%)	
Body weight (kg)	62.23 ± 18.99	65.07 ± 45.58	74.97 ± 15.48	*p* < 0.001
BMI (kg/m^2^)	20.70 ± 3.34	23.04 ± 11.65	26.51 ± 6.14	*p* < 0.001
BMI classification				*p* < 0.001
Underweight	44 (28.2)	23 (8.1%)	10 (1.4)	
Normal	98 (62.8%)	213 (75.3%)	301 (41.0)	
Overweight	10 (6.4%)	42 (14.8%)	273 (37.2)	
Obese	4 (2.6%)	5 (1.8%)	150 (20.4)	
FL total score	32.03 ± 6.64 ^a^	35.21 ± 7.31 ^b^	33.44 ± 6.62 ^a^	*p* < 0.001

BID: body image dissatisfaction, BMI: body mass index, FL: food literacy. ^a,b^: Different letters indicate statistically significant difference as a result of ANOVA.

**Table 3:** Food literacy as a mediator in the relationship between body image dissatisfaction and body mass index.

Model	Beta	SE	*p*	Bootstrap Effect %95 Cl	
				Lower Limit	Upper Limit
BID → FL	2.015	0.463	<0.001	1.106	2.923
BID → BMI	−2.457	0.533	<0.001	−3.504	−1.410
FL → BMI	−0.087	0.033	0.009	−0.153	−0.021
BID → FL → BMI	−2.281	0.536	<0.001	−3.334	−1.228
FL → BMI (Mediating effect)				−0.3702	−0.037
Constant	31.185 0.608 R^2^ = 0.158 *p* < 0.001				

BID: body image dissatisfaction, BMI: body mass index, FL: food literacy.

## Data Availability

The data presented in this study are available on reasonable request due to privacy and ethical restrictions.
